# Isolation and Functional Characterisation of a *fads2* in Rainbow Trout (*Oncorhynchus mykiss*) with Δ5 Desaturase Activity

**DOI:** 10.1371/journal.pone.0150770

**Published:** 2016-03-04

**Authors:** Noor Khalidah Abdul Hamid, Greta Carmona-Antoñanzas, Óscar Monroig, Douglas R. Tocher, Giovanni M. Turchini, John A. Donald

**Affiliations:** 1 Deakin University, School of Life and Environmental Sciences, Waurn Ponds, Geelong, Victoria, Australia; 2 University of Stirling, Institute of Aquaculture, School of Natural Sciences, Stirling, Scotland, United Kingdom; 3 Universiti Sains Malaysia, School of Biological Sciences, Penang, Malaysia; University Paris South, FRANCE

## Abstract

Rainbow trout, *Oncorhynchus mykiss*, are intensively cultured globally. Understanding their requirement for long-chain polyunsaturated fatty acids (LC-PUFA) and the biochemistry of the enzymes and biosynthetic pathways required for fatty acid synthesis is important and highly relevant in current aquaculture. Most gnathostome vertebrates have two fatty acid desaturase (*fads*) genes with known functions in LC-PUFA biosynthesis and termed *fads1* and *fads2*. However, teleost fish have exclusively *fads2* genes. In rainbow trout, a *fads2* cDNA had been previously cloned and found to encode an enzyme with Δ6 desaturase activity. In the present study, a second *fads2* cDNA was cloned from the liver of rainbow trout and termed *fads2b*. The full-length mRNA contained 1578 nucleotides with an open reading frame of 1365 nucleotides that encoded a 454 amino acid protein with a predicted molecular weight of 52.48 kDa. The predicted Fads2b protein had the characteristic traits of the microsomal Fads family, including an N-terminal cytochrome b5 domain containing the heme-binding motif (HPPG), histidine boxes (HDXGH, HFQHH and QIEHH) and three transmembrane regions. The *fads2b* was expressed predominantly in the brain, liver, intestine and pyloric caeca. Expression of the *fasd2b* in yeast generated a protein that was found to specifically convert eicosatetraenoic acid (20:4n-3) to eicosapentaenoic acid (20:5n-3), and therefore functioned as a Δ5 desaturase. Therefore, rainbow trout have two *fads2* genes that encode proteins with Δ5 and Δ6 desaturase activities, respectively, which enable this species to perform all the desaturation steps required for the biosynthesis of LC-PUFA from C_18_ precursors.

## Introduction

Long-chain (C ≥ 20) polyunsaturated fatty acids (LC-PUFA) play a variety of fundamental physiological roles in vertebrates. In fish they also have an important economic aspect, as fish are the primary source of n-3 LC-PUFA in the human diet [1]. Fish, like all vertebrates, are unable to synthesise polyunsaturated fatty acids (PUFA) *de novo*, and different teleost species have a range of capabilities in the bioconversion of dietary C_18_ PUFA to LC-PUFA [2–5]. In general, and likely resulting from adaptation and evolution, fish can be classified into two different generic groups according to their respective fatty acid metabolism: those able to bioconvert dietary C_18_ PUFA (essential fatty acids) such as linoleic acid (LA; 18:2n-6) and α-linolenic acid (ALA; 18:3n-3) into LC-PUFA; and those unable to bioconvert C_18_ PUFA into LC-PUFA, and thus requiring dietary LC-PUFA to satisfy their essential fatty acid requirements [6]. The former group is typically represented by freshwater species, low trophic level species, and the diadromous salmonids [7–12]. When the C_18_ PUFA (namely LA and ALA) are present in the diet, conversion to LC-PUFA can occur by a sequence involving alternate desaturation and elongation steps. During biosynthesis of arachidonic acid (ARA; 20:4n-6) and eicosapentaenoic acid (EPA; 20:5n-3), the pathway primarily proceeds through an initial desaturation step involving a Δ6 desaturase (for the conversion of LA and ALA to 18:3n-6 and 18:4n-3, respectively), followed by subsequent chain elongation (obtaining 20:3n-6 and 20:4n-3, respectively), and then a Δ5 desaturation (for the final biosynthesis of ARA and EPA, respectively) [13].

In mammals, the *fads1* gene encodes the Fads1 protein that has Δ5 desaturase activity, and the *fads2* gene encodes the Fads2 protein that has Δ6 desaturase activity [14], and both *fads* genes are present in amphibians, reptiles, and birds [15]. In fishes, *fads1* and *fads2* genes that encode proteins with Δ5 and Δ6 desaturase activity, respectively, are present in the chondrichthyan fish, catshark, *Scyliorhinus canicula* [15], and the predicted Fads1 and Fads2 proteins are found in the genome of the holocephalan elephant fish, *Callorhinchus milii*. However, the *fads1* gene has been lost in the teleost fishes, and only *fads2* genes have been cloned or identified in sequenced genomes [15]. In most teleost species, *fads2* encodes a protein that is a functional Δ6 desaturase [5, 12, 16–19], but there is functional plasticity in the desaturase activity in some species [15]. In zebrafish, *Danio rerio* and tilapia, *Oreochromis niloticus*, the *fads2* gene encodes a bifunctional protein that has both Δ5 and Δ6 desaturation activities [20, 21]. The Atlantic salmon, *Salmo salar*, has four *fads2* genes that encode proteins with Δ5 (one protein) and Δ6 (three proteins) desaturase activities [22, 23], respectively. Two *fads* genes are also present in rabbitfish, *Siganus canaliculatus*, which encode a bifunctional Δ5/Δ6 desaturase and a discrete Δ4 desaturase, respectively, the latter being the first report of a vertebrate Δ4 desaturase [24]. Subsequently, a further *fads2* mRNA encoding a protein with Δ4 desaturase activity was isolated and characterised in Senegalese sole, *Solea senegalensis* [25]. Recently, two *fads2* genes were cloned from pike silverside, *Chirostoma estor*, and found to have Δ5/Δ6 and Δ4 desaturase activity, respectively [26]. The discovery of Δ4 desaturases in some species of teleost fish indicated that they have the capacity to synthesise docosahexaenoic acid (DHA; 22:6n-3) directly from docosapentaenoic acid (DPA; 22:5n-3), and thus are independent of the classical “Sprecher” pathway that requires the elongation of DPA to 24:5n-3, its subsequent Δ6 desaturation for the production of 24:6n-3, and then a peroxisomal chain-shortening for the final synthesis of DHA [24]. Finally, Fads2 with Δ6 desaturase activity from teleosts, and especially from marine species, also show Δ8 desaturase activity [27–29], again illustrating the functional plasticity of teleost Fads2. The Δ8 activity allows for a different and parallel pathway for the bioconversion of LA and ALA into 20:3n-6 and 20:4n-3, respectively, via their elongation followed by a Δ8 desaturation, rather than the traditional Δ6 desaturation and subsequent elongation.

Rainbow trout, *Oncorhynchus mykiss*, are cultured commercially worldwide and they are commonly fed diets containing LC-PUFA, as these diets are important for the growth and health of the fish and they meet consumer expectations for the nutritional quality and health value of cultured fish products [30–34]. Currently, fish oil is the primary source of LC-PUFA in formulated feed for fish (aquafeed) [1, 10]. However, the finite and limited supply and increasing price of fish oil, have dictated that the aquafeed industry develop alternative strategies, with plant (vegetable) oils that contain no LC-PUFA being the primary option for fish oil substitution in aquafeed [1, 35–41]. The reduced levels of n-3 LC-PUFA in feeds is reflected in a reduction of EPA and DHA in fish tissues, even in species with documented capabilities of bioconverting dietary ALA into n-3 LC-PUFA, such as rainbow trout [42, 43], which can have important consequences for human consumption.

Given the importance of LC-PUFA composition and metabolism in fish for nutritional reasons, it is essential to have a full understanding of the enzymes involved in the biosynthetic pathway(s). Previously, a *fads2* was isolated from rainbow trout and the encoded protein shown to possess Δ6 desaturase activity [5]. The present study reports the isolation, cloning and characterisation of a second *fads2* cDNA (termed *fads2b*) in rainbow trout that encodes a protein with Δ5 desaturase activity. The findings provide new information about endogenous n-3 LC-PUFA biosynthesis in a commercially important teleost fish species.

## Materials and Methods

### Animals and tissue collection

Animal experimentation was approved by the Deakin University Animal Ethics Committee (AEC approval A41-2011). Rainbow trout, *O*. *mykiss*, were obtained from the Department of Primary Industries (Snobs Creek, Victoria, Australia) and housed in a recirculating aquaculture system (RAS) located at Deakin University, Warrnambool, Australia. The aquaculture system was equipped with an in-line oxygen generator and a physical and biological filtration plant. Water temperature (15.0 ± 0.8°C), pH (8.26 ± 0.04), and oxygen concentration (9.14 ± 0.30 mg.L^−1^) were monitored daily. The dissolved ammonia and nitrite levels were measured weekly using Aquamerck test kits (Merck, Darmstadt, Germany), and were maintained below 0.20 and 0.25 mg.L^−1^, respectively. The system operated on a 12:12 h light-dark cycle. All fish were fed a commercial trout diet (40% protein, 20% lipid, closed formula, Ridley Aquafeed, Australia). For experimentation, fish were humanely killed using an overdose of anaesthetic (10% AQUI-S; AQUI-S New Zealand Ltd., Lower Hutt), and the gill, liver, muscle, adipose tissue, brain, heart, intestine, kidney, pyloric caeca and stomach tissues were snap frozen in liquid nitrogen and stored at –80°C until required.

### RNA isolation and cloning of rainbow trout *fads2b* cDNA

**Fig 1:**
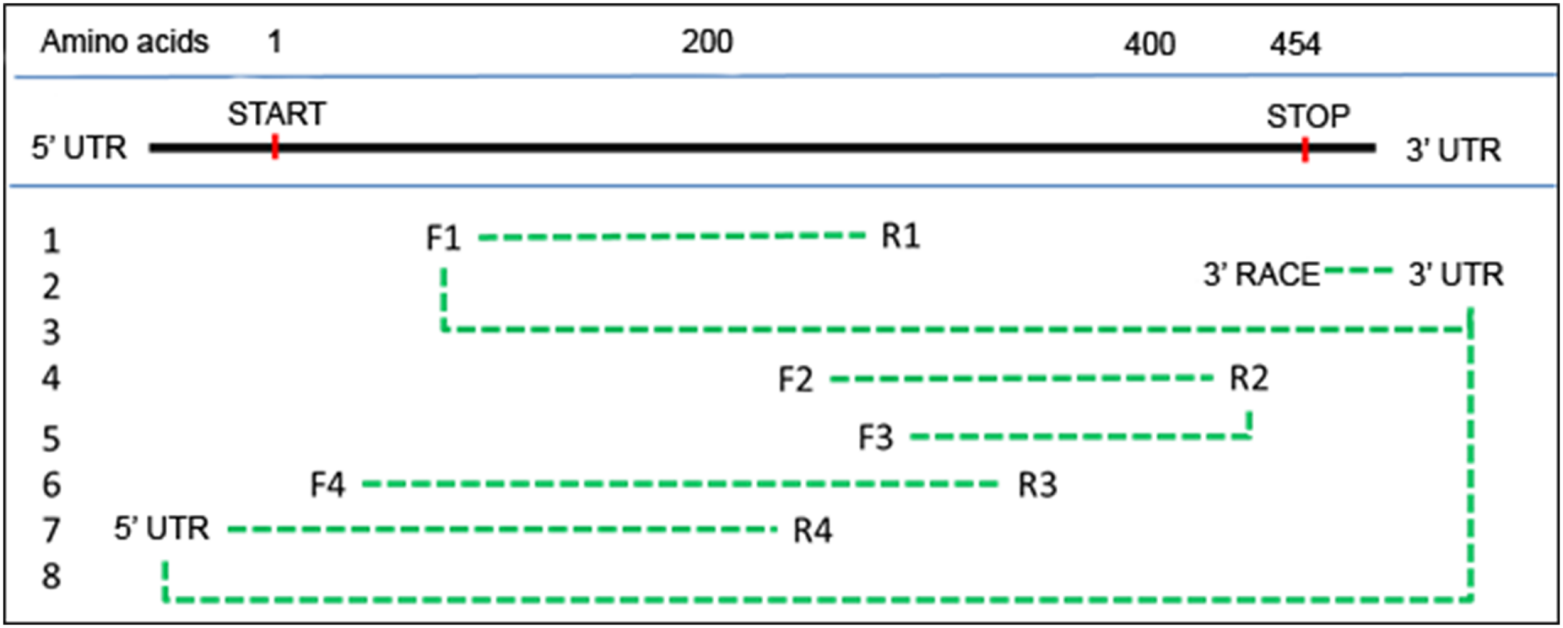
Diagram showing the sequential steps (numbers 1 to 8 on left side of Figure) for cloning of a rainbow trout *fads2b* cDNA using salmon *fads2b*/Δ5 as a template. The primer sets for the eight PCR fragments are listed in Table 1. The rationale of each step is as follows. **Step 1**: initial amplification (672 bp) of trout *fads2b*; **Step 2**: 3´ RACE PCR to amplify (201 bp) from 3´ end of ORF and UTR of trout *fads2b*; **Step 3**: amplification (1349 bp) of trout *fads2b* using sequence obtained in steps 1 and 2 for primer design; **Steps 4 and 5**: confirmation of trout *fads2b* compared to trout *fads2*/Δ6 (step 4 593 bp, step 5 470 bp); **Steps 6 and 7**: amplification of 5´ end of trout *fads2b* ORF and UTR sequences, respectively (step 6 593 bp, step 7: 470 bp); **Step 8**: amplification (1578 bp) of full trout *fads2b* mRNA sequence.

The liver was chosen for the isolation of rainbow trout *fads2b* mRNA as it is the predominant site for fatty acid synthesis in vertebrates. Briefly, approximately 20 mg of liver was added to a 1.5 mL centrifuge tube containing 10 1.0 mm silica beads (Biospec Products, Inc.) and 1 mL of TriReagent (Sigma). The tissue was mechanically disrupted using a high-speed bench top homogeniser (FastPrep24), at a speed of 6.5 M/sec for 60 sec. Isolation of total RNA was then performed according to the manufacturer’s protocol for TriReagent. For cDNA synthesis, 3 μg of RNA was reverse transcribed into first strand cDNA using oligo (dT) priming and SuperScriptII Reverse Transcriptase (Invitrogen), according to the manufacturer’s protocol. The quality of cDNA was verified by the expression of *β-actin* as a control mRNA (accession number: AF157514). Primers for amplification of rainbow trout *fads2b* were designed using Gene Runner v.3.05 and the Atlantic salmon (*S*. *salar*) sequence for *fads*2/Δ5 (21) as a reference sequence (NCBI database, accession number: AF478472). The primers used for amplifying rainbow trout *fads2b* cloning are listed in Table 1, and the PCR strategy is illustrated in Fig 1, with each step described in the legend to the figure.

**Table 1:** List of primers for RT-PCR, 3´ RACE, and cloning of the *fads2b* coding sequence into pYES2 vector.

	Primer sequence and amplification (5´--- 3´)
	*cDNA cloning*
F1	GGAGAAGATGCCACGGAAG
F2	CCTGATATCAACTCACTACAT
F3	CCACTAATCGTTCCAGTGTTTTT
F4	CGGGCTTGAGCCCGATGGA
R1	AAACATGGTCCGGAATATCT
R2	CTGACAACATCAGTCATGCCTTT
R3	CTCATCGACCACGCCAGAT
R4	CCAATGACAAACTTGTGCAGTT
	*3*´ *RACE*
3'RACE	ATGACTGATGTTGTCAGGTCA
	*Untranslated region*
5'UTR	AACGCTGTCTGGAAAACATCTC
3'UTR	CTGACAAGGATTAAAACAAT
	*Rainbow trout fads2b mRNA expression*
Tr*fads2b*F	TTCCGGACCATGTTTTCACA
Tr*fads2b*R	ACAATCATGTTTGCAGCGAT
	*Rainbow trout fads2b primers with restriction sites*
*Bam* H1	ATTGGATCCAGGATGGGGGGCGGAGG
*Xho* I	TAACTCGAGGATTTATTTATGGAGATACGCATC

Standard PCR amplification was performed in 0.2 mL thin-walled tubes using an Eppendorf Mastercycler. The 20 μL reaction mixture contained: 1 X buffer, 0.25 mM dNTP, 1.875 mM MgCl_2_, 1 μM of each primer, 1 μL of cDNA, and 2 units of Taq polymerase. PCR was conducted under the following conditions: an initial denaturation at 94°C for 3 min, followed by 30 to 36 cycles of denaturation at 94°C for 15 sec, annealing at 58–60°C for 30 sec, and extension at 72°C for 3 to 3.5 min, and a final extension step of 72°C for 5 min. PCR products were visualised using a Syngene gel documentation system (Synoptics Ltd, UK), were then excised, and then purified using a NucleoSpin Extract II kit (Scientifix) according to the manufacturer’s protocol. Nucleotide sequences were determined by standard dye terminator chemistry using a BigDye terminator v3.1 cycle sequencing kit (Applied Biosystems) and an ABI PRISM 3100 Genetic Analyser (Deakin University).

A 3´ rapid amplification of cDNA ends (3´ RACE) method was used to obtain the 3´ untranslated region (UTR) of rainbow trout *fads2b* using the 3´ full RACE core set (Takara). Reverse transcription of the mRNA was performed according to the manufacturer’s protocol, with 1 μL of cDNA used as a template in the first round of PCR amplification. The reaction was performed in a total volume of 20 μL and contained: 1 x PCR buffer, 0.2 mM dNTPs, 2.5 mM MgCl_2_, 1 μM of gene-specific reverse primer (Table 1), 0.5 μM 3' end PCR primer, and 2 units of Taq polymerase. The 3´-RACE PCR reactions had an initial denaturation at 94°C for 3 min, followed by 40 cycles of denaturation at 94°C for 45 sec, annealing at 50–58°C for 30 sec, and extension at 72°C for 45 sec, with a final extension step at 72°C for 3 min. A nested PCR reaction was performed using 1 μL of double stranded cDNA from the first PCR reaction, following the same PCR condition as the first round PCR.

### Tissue distribution of rainbow trout *fads2b* mRNA

The expression of rainbow trout *fads2b* mRNA was determined in adipose tissue, brain, gill, heart, intestine (hind gut), kidney, liver, muscle, pyloric caeca, and stomach from three animals. RNA isolation, cDNA synthesis, and PCR were performed as described above using the PCR primers detailed in Table 1. The PCR amplification was performed for 36 cycles and *β-actin* mRNA expression was used to validate the quality of the synthesised cDNA.

### Sequence and phylogenetic analysis

The full-length sequences of rainbow trout *fads2b* and the predicted Fads2b protein were analysed using the Basic Local Alignment Search Tool (BLAST, www.ncbi.nlm.nih.gov/blast/; [44]). The molecular weight of the predicted rainbow trout Fads2b protein was determined using the tools available at http://www.bioinformatics.org/sms/prot_mw.html. Alignment of rainbow trout Fads2b with Fads2/Δ5 desaturase and Fads2/Δ6 desaturases from Atlantic salmon and Fads1/Δ5 and Fads2 Δ6 from human was performed using the GeneDoc program and SOSUI software (http://bp.nuap.nagoya-u.ac.jp/sosui/sosui).

For phylogenetic analysis, Fads sequences were obtained from the NCBI GenBank database (www.ncbi.nlm.nih.gov/Genbank/index.html) (see S1 Table), and aligned using Clustal Omega (http://www.ebi.ac.uk/Tools/msa/clustalo/; [45]). A maximum likelihood tree was constructed in MEGA6 using the Jones-Taylor-Thornton matrix based method for amino acid substitutions. The statistical significance of each branch was evaluated by bootstrapping with 1000 replicates.

### Heterologous expression of rainbow trout *fads2b* in yeast

The rainbow trout *fads2b* coding sequence was obtained by PCR from full-length cDNA with primers containing *Bam* HI and *Xho* I restriction sites, respectively (Table 1). This fragment and the yeast galactose-induced expression plasmid pYES2 (Invitrogen, UK) were digested with the corresponding restriction endonucleases (New England BioLabs, Herts, UK), and ligated using T4 DNA Ligase (Bioline, London, UK). The resulting plasmid construct pYES2-*fads2b* was transformed into *Saccharomyces cerevisiae* (strain INVSc1) using the S.C. EasyComp Transformation kit (Invitrogen) and incubated for 3 days at 30°C in yeast drop-out plates ^-uracil^. A single colony of transgenic yeast was grown in *S*. *cerevisiae* minimal medium ^-uracil^ (SCMM^-ura^) for 24 h prior supplementation with 2% D-galactose and one of the following fatty acid substrates diluted in an aqueous detergent solution: ALA, LA, eicosatrienoic acid (20:3n-3), eicosadienoic acid (20:2n-6), eicosatetraenoic acid (20:4n-3), dihomo-γ-linolenic acid (20:3n-6), docosapentaenoic acid (22:5n-3) and adrenic acid (22:4n-6). Substrate FA final concentrations were 0.5 mM for C18, 0.75 mM for C20 and 1 mM for C22 fatty acids. The fatty acid substrates (> 99% pure) and chemicals used to prepare the SCMM^-ura^ were purchased from Sigma Chemical Co. Ltd. After 2 days, yeast were harvested and washed with Hank’s balanced salt solution containing 1% fatty acid-free bovine serum albumin prior to lipid extraction and fatty acid analyses. Yeast transformed with pYES2 containing no insert were cultured under the same conditions described above and used as control treatments. Yeast fatty acid analysis was performed as described previously [9, 12].

## Results

### Sequencing of rainbow trout *fads2b* mRNA

**Fig 2:**
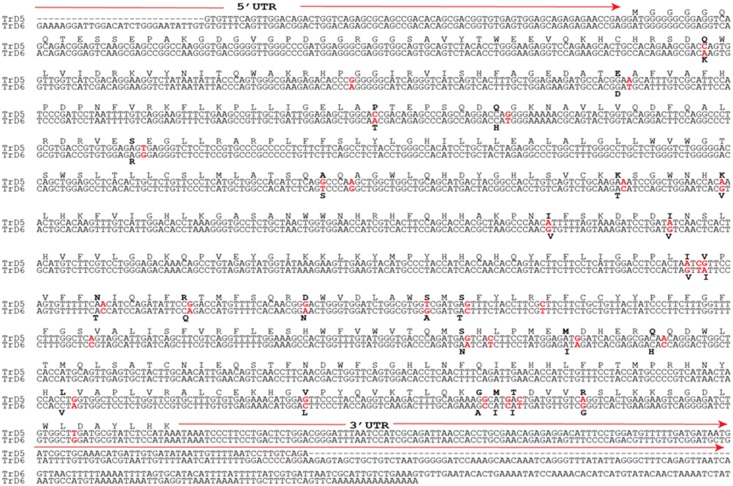
An alignment of the nucleotide and amino sequences of rainbow trout Fads2b (accession number: JQ087459) with those of Fads2a from trout (accession number: AF301910). The differences in the sequences are indicated in red for nucleotides and bold for amino acids. The untranslated regions (UTR) are marked by solid red arrows above the sequence.

**Fig 3:**
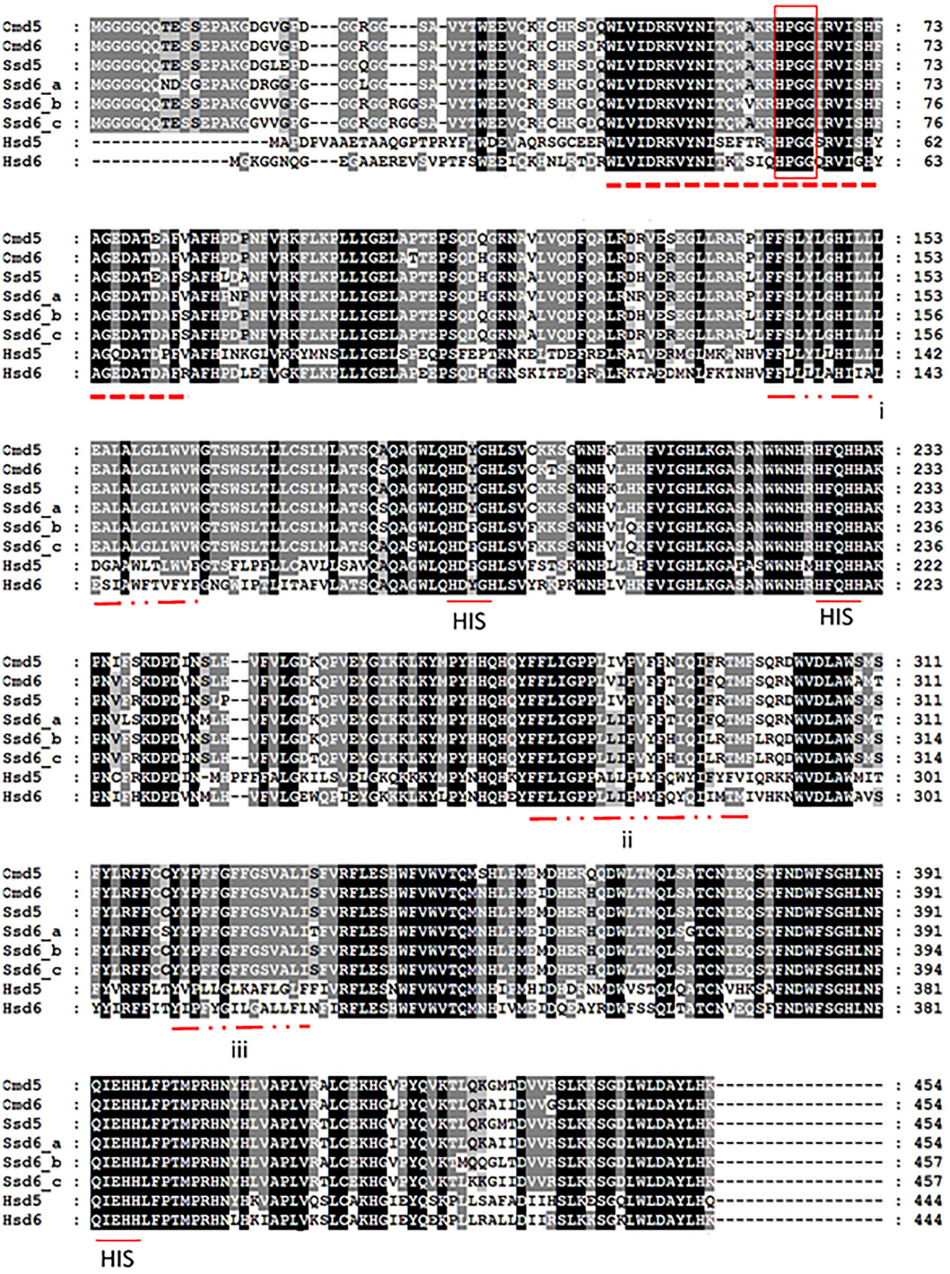
Alignment of the rainbow trout (Om) Fads2b/Δ5 deduced amino acid sequence against Fads2/Δ6 from rainbow trout, Fads2/Δ5 desaturase and Fads2/Δ6 desaturases from Atlantic salmon (Ss), and Fads1/Δ5 and Fads22/Δ6 from human (Hs). Black, dark grey and grey shaded area indicates 100%, 80%, and 60% conserved regions, respectively, using the GeneDoc program. The dotted line denotes the cytochrome b5 domain, and a heme-binding motif is boxed. Three transmembrane regions (double dot-dash lined) are predicted by SOSUI software (http://bp.nuap.nagoya-u.ac.jp/sosui/sosui). Histidine boxes are underlined.

Overlapping fragments of a putative rainbow trout *fads2b* cDNA were amplified using standard and 3´-RACE PCR, which were assembled to construct a consensus sequence. The full-length putative rainbow trout *fads2b* cDNA contained 1578 nucleotides with an open reading frame (ORF) of 1365 base pairs that encoded a 454 amino acid protein, with 74 nucleotides in the 5´ UTR and 139 nucleotides in the 3´ UTR (Fig 2). The molecular weight of the predicted protein was 52.48 kDa. The predicted rainbow trout Fads2b protein had the typical traits of the microsomal fatty acyl desaturase family, including an N-terminal cytochrome b5 domain containing the heme-binding motif (HPPG), histidine boxes (HDXGH, HFQHH and QIEHH) and three transmembrane regions (Fig 3). The predicted rainbow trout Fads2b protein shared 94.2% identity with rainbow trout Fads2a/Δ6 (accession number: NP_001117759) and 95% identity to Fads2b of Atlantic salmon (accession number: NP_001117014.1), but was most similar (98.5%) to Fads2a of masu salmon, *Oncorhynchus masou*, (accession number: BAB63440). The rainbow trout *fads2b* mRNA and predicted protein sequence were deposited in the GenBank database (accession number: AFM77867).

### Phylogenetic analysis

**Fig 4:**
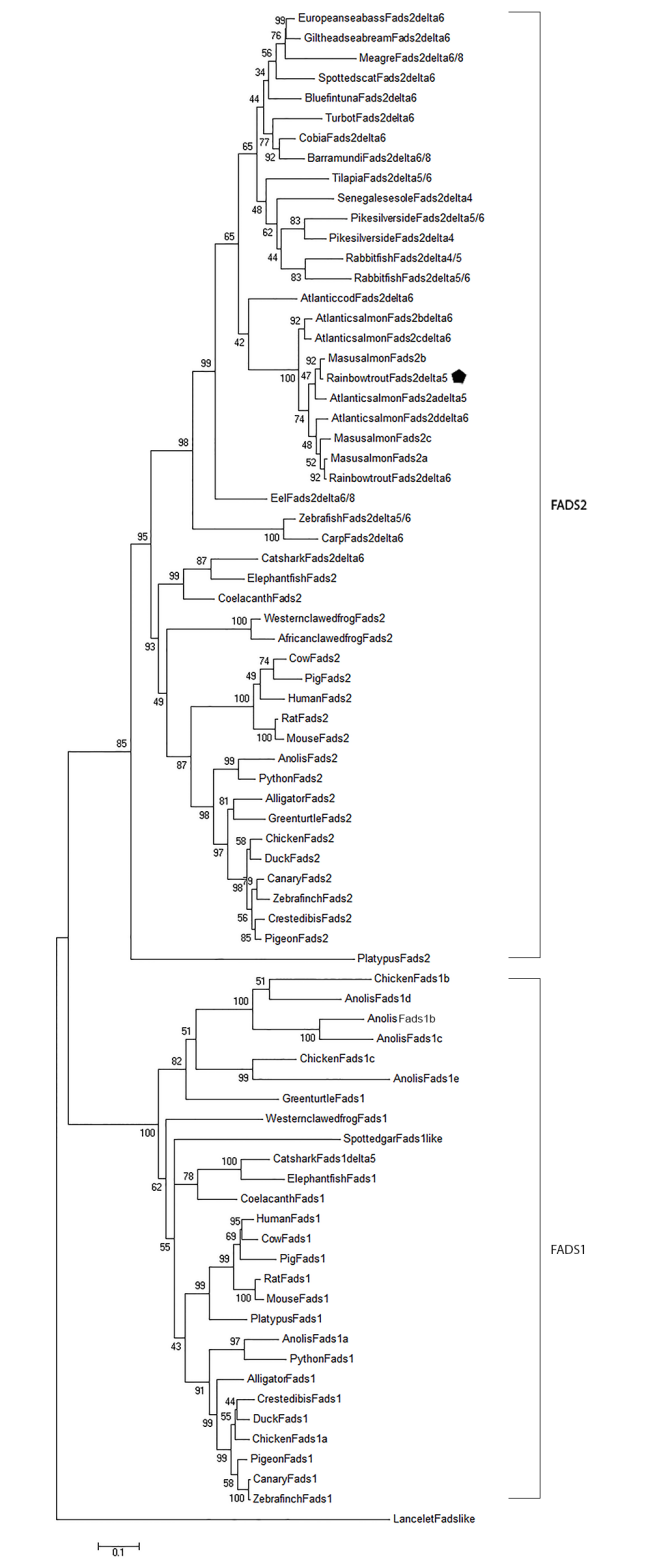
Phylogenetic analysis of vertebrate Fads proteins as inferred using the Neighbour-Joining method applying a maximum likelihood approach. The position of rainbow trout Fads2b/Δ5 is marked.

A neighbour-joining tree showing the phylogenetic relationship of vertebrate Fads proteins is shown in Fig 4. The vertebrate Fads proteins clustered into two primary clades, Fads1 and Fads2, with good bootstrap support. As expected, the rainbow trout Fads2a protein grouped with other salmonid Fads2 proteins.

### Tissue distribution of rainbow trout *fads2b* mRNA

**Fig 5:**
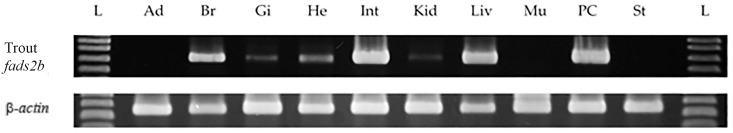
The expression pattern of *fads2b* mRNA in different tissues of rainbow trout as determined by conventional RT-PCR for 36 cycles. A partial *β-actin* mRNA was used as an internal standard. A similar *fads2b* mRNA distribution profile was obtained in three animals. Ladder L, Adipose tissue Ad, Brain Br, Gill Gi, Heart He, Intestine Int, Kidney Kid, Liver Liv, Muscle Mu, Pyloric caeca PC, Stomach St.

The expression profile of *fads2b* mRNA in rainbow trout tissues is shown in Fig 5. Although comparisons of mRNA expression from RT-PCR analyses have to be made cautiously, some patterns can be observed in the tissue distribution of *fads2b* transcripts. A relatively higher *fads2b* mRNA expression was found in the liver, intestine, pyloric caeca, and brain. A relatively lower *fads2b* mRNA expression was observed in the gill, heart and kidney. No expression was found in the stomach, muscle and adipose tissue. A similar expression profiles was found in three animals.

### Functional characterisation of rainbow trout Fads2b in yeast

**Fig 6:**
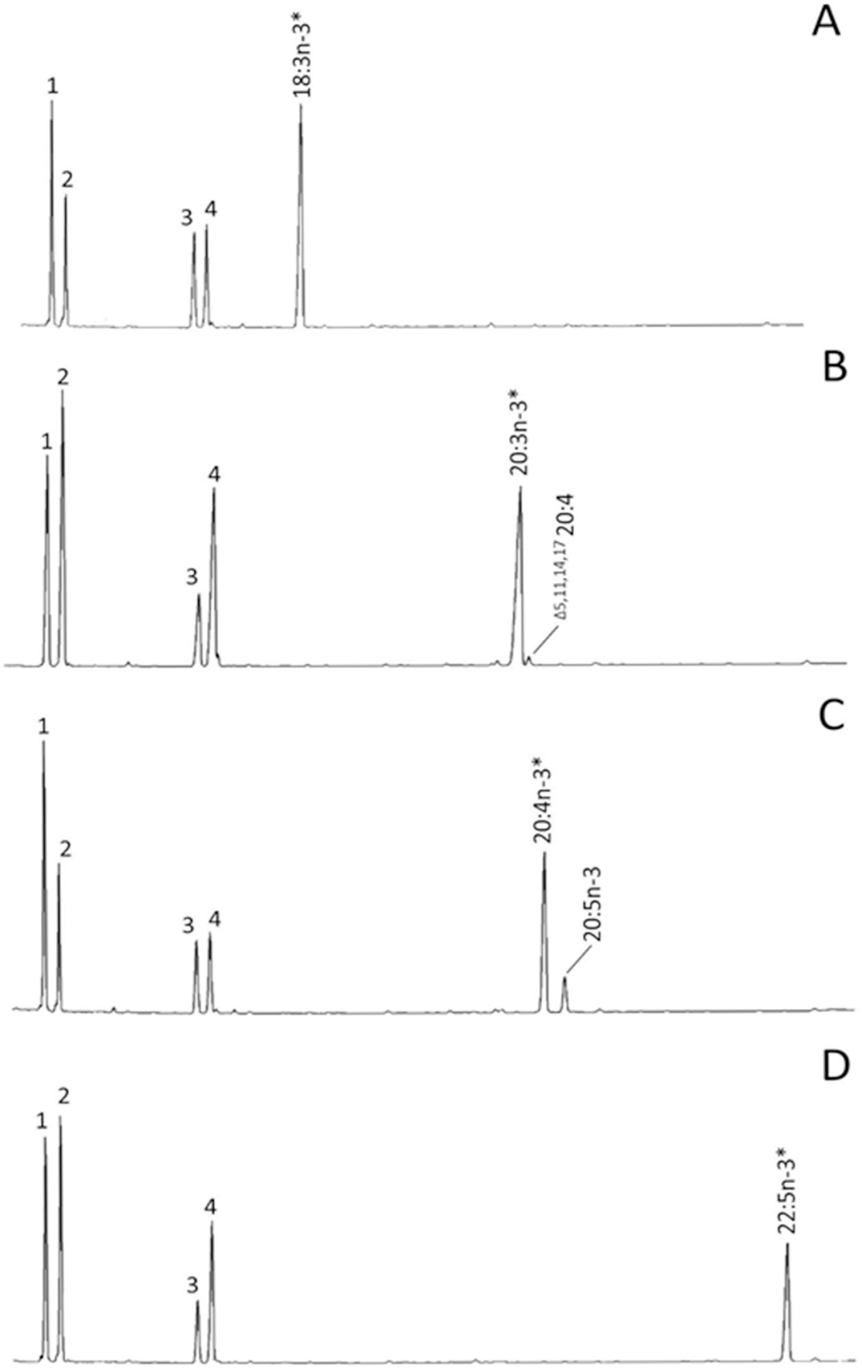
Functional characterisation of the newly cloned rainbow trout Fads2b in yeast (*Saccharomyces cerevisiae*). The fatty acid profiles of yeast transformed with pYES2 containing the coding sequence of the rainbow trout *fads2b* as an insert, were determined after the yeast was grown in the presence of one of the exogenously added substrates 18:3n-3 (A), 20:3n-3 (B), 20:4n-3 (C) and 22:5n3 (D). Peaks 1–4 in panels A-D are the main endogenous fatty acids of *S*. *cerevisiae*, namely 16:0 (1), 16:1 isomers (2), 18:0 (3) and 18:1n-9 (4). Additionally peaks derived from exogenously added substrates (“*”) and desaturated products are indicated accordingly in panels A-D. Vertical axis, flame ionisation detector response; horizontal axis, retention time.

The ability of rainbow trout Fads2b to desaturase PUFA of the n-3 and n-6 series was determined by quantification of the relative fatty acid conversions obtained when transformed *S*. *cerevisiae* containing either the empty pYES2 vector (control), or a vector with the rainbow trout *fads2b* ORF insert grown in the presence of potential fatty acid substrates. For this, yeast transformed with pYES2-Fads2b were grown in the presence of Δ6 substrates (18:3n-3, 18:2n-6), Δ8 substrates (20:3n-3 and 20:2n-6), Δ5 substrates (20:4n-3 and 20:3n-6) and Δ4 substrates (22:5n-3 and 22:4n-6). The fatty acid composition of the yeast transformed with empty pYES2 (control) showed the main fatty acids normally found in *S*. *cerevisiae*, namely 16:0, 16:1 isomers, 18:0 and 18:1n-9, and whichever exogenous PUFA was added (data not shown). This is consistent with this yeast strain not possessing any PUFA desaturation capabilities (12). However, based on GC retention time and confirmed by GC-MS, rainbow trout Fads2b showed significant desaturation activity towards the Δ5 substrate 20:4n-3 converting it to EPA, which accounted for over 17% of the total substrate conversion (Fig 6), whereas the n-6 Δ5 substrate 20:3n-6 was desaturated to a lower extent (2% conversion) (Table 2). Additional peaks were not observed with any of the exogenously added Δ6 or Δ4 fatty acid substrates. As reported for other teleost species, including the previously described rainbow trout *fads2* Δ6 paralog, the newly characterised Fads2 displayed more activity towards n-3 PUFA than n-6 PUFA (Table 2) (5). It was interesting to note that 20:2n-6 (^Δ11,14^ 20:2) and 20:3n-3 (^Δ11,14,17^ 20:3) were desaturated in position Δ5 to produce the non-methylene interrupted (NMI) fatty acids ^Δ5,11,14^ 20:3 and ^Δ5,11,14,17^ 20:4, respectively (Table 2; Fig 6).

**Table 2:** Functional characterisation of the putative rainbow trout Fads2b in yeast.

Substrate	Activity	Product	% Conversion
18:3n-3	Δ6	18:4n-3	0.0
18:2n-6	Δ6	18:3n-6	0.0
20:3n-3	Δ5 (not Δ8)	^Δ5,11,14,17^ 20:4	2.6
20:2n-6	Δ5 (not Δ8)	^Δ5,11,14^ 20:3	1.1
20:4n-3	Δ5	20:5n-3	17.8
20:3n-6	Δ5	20:4n-6	2.1
22:5n-3	Δ4	22:6n-3	0.0
22:4n-6	Δ4	22:5n-6	0.0

## Discussion

Rainbow trout had previously been reported to have no dietary requirement for preformed dietary n-3 LC-PUFA, and to be able to bioconvert ALA into EPA and DHA, and that this bioconversion was relatively efficient and followed the so-called “Sprecher pathway” [7, 8, 41, 42]. As discussed above, the majority of teleost fish have a single *fads2* gene that encode primarily monofunctional Δ6 desaturase proteins [5, 16–19]. However, some species deviate from this typical pattern. For example, Atlantic salmon, zebrafish, Senegalese sole, pike silverside, barramundi and rabbitfish have *fads2* genes that encode Fads2 proteins with functionalities that include Δ4, Δ5 and Δ8 [12, 21–25, 29]. Prior to the current study, the only documented and known *fads2* gene encoding a discrete monofunctional Δ5 desaturase protein in a teleost fish was that of Atlantic salmon [21]. The presence of a Fads-like enzyme with Δ5 desaturase activity in rainbow trout had been indicated indirectly by *in vivo* trials [46], but the protein had not been isolated or characterised. In the present study, a *fads*2 gene that encodes a Δ5 desaturase has been isolated and functionally characterised in rainbow trout for the first time. Thus, rainbow trout follow the same pattern previously reported in Atlantic salmon and have the capacity to synthesise EPA and ARA from ALA and LA, respectively, through separate *fads2* genes that encode proteins with Δ6 and Δ5 desaturase activity, respectively. The confirmation of a monofunctional Δ5 desaturase protein in rainbow trout is important considering the commercial value of this species, the issues surrounding the provision of preformed dietary n-3 LC-PUFA (i.e. fish oil) in commercially aquaculture species, and in consideration of our understanding of vertebrate fatty acid metabolism and its evolution.

As expected, the phylogenetic analysis of vertebrate Fads protein grouped the deduced rainbow trout Fads2b protein with other salmonid Fads2 proteins. Interestingly, the two salmonid Fads2 proteins that show Δ5 desaturase activity are no more similar to each other than to the other salmonid Δ6 desaturase proteins. The evolution of the *fads* genes in vertebrates has recently been investigated [15]. It was proposed that the ancestral gnathostome possessed *fads1* and *fads2* genes, as they are both found in chondrichthyans and have been characterised as Δ5 and Δ6 desaturases, respectively, in catshark [15]; both *fads1* and *fads2* genes are also found in the coelacanth genome. As all teleost fish species examined to date only possess the *fads2* gene, Castro and colleagues [15] proposed that the *fads1* gene must have been lost in the actinopterygian lineage that gave rise to teleost fishes. However, analysis of the annotated genome of the non-teleost actinopterygian, the spotted gar fish, *Lepisosteus oculatus*, reveals *fads* genes that encodes proteins with homology to Fads1; the functional characterisation of spotted gar Fads proteins would be of interest.

Rainbow trout *fads2b* mRNA was more highly expressed in the liver, intestine including pyloric caeca, and brain compared to other tissues, which was consistent with previous studies that demonstrated these tissues are important sites of LC-PUFA biosynthesis in salmonid fish [47–49]. A high expression of *fads2b* mRNA in the brain of rainbow trout is consistent with the expression pattern of *fads2* (Δ6) in rainbow trout [50], and *fads2* (Δ5) [23] and the three *fads2* (Δ6) genes in Atlantic salmon [12]. Furthermore, many other studies have shown that teleost brain expresses genes encoding the LC-PUFA biosynthesis enzymes including Fads and elongation of very long-chain fatty acid proteins (Elovl; see [3,16, 27, 49, 51–53]). Interestingly, it appears that the expression of *fads* genes is higher in the brain of marine species compared with freshwater species [54]. However, this pattern is not found in euryhaline species such as rabbitfish [54], and the marine Senegalese sole [55] in which the *fads* mRNA expression was higher in the liver compared to the brain. The high expression of *fads2* genes in the brain of teleosts could relate to the significance of LC-PUFA in the functionality of neuronal tissue, which has been reported in mammals [56–58]. In rainbow trout, the high expression of *fads2b* mRNA in the pyloric caeca is again consistent with that of *fads2* (Δ6) in rainbow trout [50] and the salmon *fads* genes [12]. In fact, in Atlantic salmon, the pyloric caeca had the highest mRNA expression of *fads2* (Δ6) and *fads2* (Δ5), which reflects a high LC-PUFA biosynthesis in this tissue [49, 59]. In addition, the pyloric caeca expresses *Elovl* mRNA and the transcription factors, liver x receptor (*Lxr*) and sterol regulatory element-binding protein (*Srebp*), the latter being important in the control of lipid and fatty acid metabolism by regulating *fads* mRNA expression [60, 61]. Thus, the pyloric caeca contains the appropriate intracellular machinery for LC-PUFA biosynthesis [12, 52, 60, 62].

Commonly, the conventional pathway for synthesising LC-PUFA involves the activity of Δ6 and Δ5 desaturases. However, in addition to the action of Δ6 and Δ5 in LC-PUFA biosynthetic pathways, an alternative pathway has been identified in fish involving the action of Fads2 as a Δ8 desaturase [27]. The trout Fads2 characterised in the present study did not show any Δ8 desaturase activity, as both eicosadienoic (20:2n-6) and eicosatrienoic (20:3n-3) acids, rather than being Δ8 desaturated to 20:3n-6 and 20:4n-3, respectively, were converted into the NMI fatty acids ^Δ5,11,14^ 20:3 and ^Δ5,11,14,17^ 20:4, respectively. Nevertheless, it is very unlikely that the biosynthesis of NMI fatty acids observed in the yeast expression systems would occur *in vivo*, as availability of these substrates is limited and the possible presence of the other Fads2 with Δ8 activity like the previously characterise desaturase [26] would allow 20:2n-6 and 20:3n-3 to be incorporated into the conventional pathway.

In conclusion, this study provides the first report of a *fads2* gene that encodes a protein with monofunctional Δ5 activity from rainbow trout. Understanding of the role of Δ5 desaturase in fish provides information regarding the evolutionary biology of lipid metabolism and is also relevant for the advancement of novel aquafeed products in commercially important fish species. Although there has been significant interest in the use of plant-derived ingredients in aquafeeds as an alternative to marine resources, the ability to convert plant-derived C_18_ PUFA, ALA and LA, to C_20/22_ LC-PUFA in fish shows considerable variation between species. Further study on *fads2b* expression in the brain of rainbow trout would provide supplementary evidence regarding the significance of the brain in LC-PUFA biosynthesis. In addition, further elucidation of the biosynthetic pathways regulating lipid metabolism in fish will be required to produce a commercially profitable product containing high levels of n-3 LC-PUFA while also reducing the dependency on wild-caught marine ingredients for feeds.

## Supporting Information

S1 Table List of common names and accession numbers of the vertebrate species used to generate the phylogenetic tree of Fads1 and Fads2 proteins shown in Fig 4. (DOCX)
