# Supplementary material for: Isolation and Functional Characterisation of a fads2 in Rainbow Trout (Oncorhynchus mykiss) with Δ5 Desaturase Activity
Source: PLoS One. 2016 Mar 4;11(3):e0150770. doi: 10.1371/journal.pone.0150770 (PMC4778901; doi:10.1371/journal.pone.0150770)
Supplement: S1 Table — (DOCX) [file pone.0150770.s001.docx]

**S1 Table**: List of common names and accession numbers of the vertebrate species used to generate the phylogenetic tree of Fads1 and Fads2 proteins shown in Fig. 4

| **Fads1** | **Fads2** |
| --- | --- |
| **Mammals**  Human O60427.3  Pig NP_001106512  Rat NP_445897  Mouse AAH26848  Cow XP_612398.4  Platypus XP_007671143 | **Mammals**  Human AAG23121  Pig NP_001165221  Rat NP_112634  Mouse NP_062673  Cow NP_001076913  Platypus XP_007664805 |
| **Birds**  Chicken 1a XP_421052  Chicken 1b XP_426408  Chicken 1c XP_421051  Atlantic canary XP_009095332  Crested ibis XP_009459763  Duck XP_005024318  Zebrafinch XP_002194926  Pigeon XP_005511095 | **Birds**  Chicken NP_001153900  Atlantic canary XP_009095330.  Crested ibis XP_009459650  Duck XP_005024317  Zebrafinch XP_002194944.2  Pigeon XP_005511097 |
| **Reptiles**  Green anole 1a XP_003224167  Green anole 1b XP_003224189  Green anole 1c XP_003224188  Green anole 1d XP_003224187  Green anole 1e XP_003224186  American alligator XP_006274989  Burmese python XP_007426604  Green sea turtle EMP32024 | **Reptiles**  Green anole XP_003224168  American alligator XP_006274951  Burmese python XP_007426603  Green sea turtle XP_007063933 |
| **Amphibians**  Western clawed frog XP_002943012.2 | **Amphibians**  Western clawed frog NP_001120262  African clawed frog NP_001086853 |
| **Teleost fishes** | **Teleost fishes**  Zebrafish ∆5/6 AF309556  Cobia ∆6 ACJ65149  Barramundi ∆6/8 ACS91458  European sea bass ∆6 ACD10793  Gilthead sea bream ∆6 AAL17639  Turbot ∆6 AAS49163  Carp ∆6 AF309557_1  Eel ∆6/8 ACI32415  Bluefin tuna ∆6 ADG62353  Spotted scat ∆6 AHA62794  Atlantic cod ∆6 AAY46796  Meagre ∆6/8 AGG69480  Tilapia ∆5/6 AGV52807.1  Pike silverside ∆5/6 AHX39207  Pike silverside ∆4 AHX39206  Senegalese sole ∆4 AEQ92868  Rabbitfish ∆4 ADJ29913  Rabbitfish ∆6\|ABR12315.2  Masu salmon 2a/∆5-like ABU87822  Masu salmon BAB63440  Masu salmon BAB71963  Rainbow trout ∆6 NP_001117759  Rainbow trout ∆5 AFM77867  Atlantic salmon 2a/∆5 NP_001117014 Atlantic salmon 2b/∆6 NP_001165251  Atlantic salmon 2c/∆6 NP_001165752  Atlantic salmon 2d/∆6 NP_001117047 |
| **Non-teleost fishes**  Spotted gar XP_006642809.1  Cat shark AEY94454  Coelacanth XP_005988035.1  Elephant fish XP_007885635.1 | **Non-teleost fishes**  Catshark AEY94455  Coelacanth XP_005988034.1  Elephant fish XP_007885636.1 |
